# Algevir: An Expression System for Microalgae Based on Viral Vectors

**DOI:** 10.3389/fmicb.2017.01100

**Published:** 2017-06-30

**Authors:** Bernardo Bañuelos-Hernández, Elizabeth Monreal-Escalante, Omar González-Ortega, Carlos Angulo, Sergio Rosales-Mendoza

**Affiliations:** ^1^Laboratorio de Biofarmacéuticos recombinantes, Facultad de Ciencias Químicas, Universidad Autónoma de San Luis PotosíSan Luis Potosí, Mexico; ^2^Sección de Biotecnología, Centro de Investigación en Ciencias de la Salud y Biomedicina, Universidad Autónoma de San Luis PotosíSan Luis Potosí, Mexico; ^3^Laboratorio de Bioseparaciones, Facultad de Ciencias Químicas, Universidad Autónoma de San Luis PotosíSan Luis Potosí, Mexico; ^4^Grupo de Inmunología & Vacunología. Centro de Investigaciones Biológicas del Noroeste, SC., Instituto Politécnico Nacional 195La Paz, Mexico

**Keywords:** *Schizochytrium* sp., marine microalgae, vaccine, replicon, viral elements, geminivirus, recombinant protein yield, transient expression

## Abstract

The use of recombinant algae for the production of valuable compounds is opening promising biotechnological applications. However, the development of efficient expression approaches is still needed to expand the exploitation of microalgae in biotechnology. Herein, the concept of using viral expression vectors in microalgae was explored for the first time. An inducible geminiviral vector leading to Rep-mediated replication of the expression cassette allowed the production of antigenic proteins at high levels. This system, called Algevir, allows the production of complex viral proteins (GP1 from *Zaire ebolavirus*) and bacterial toxin subunits (B subunit of the heat-labile *Escherichia coli* enterotoxin), which retained their antigenic activity. The highest achieved yield was 1.25 mg/g fresh biomass (6 mg/L of culture), which was attained 3 days after transformation. The Algevir system allows for a fast and efficient production of recombinant proteins, overcoming the difficulties imposed by the low yields and unstable expression patterns frequently observed in stably transformed microalgae at the nuclear level; as well as the toxicity of some target proteins.

## Introduction

The use of microalgae for the production of valuable proteins is acquiring relevance in the biotechnology field. Several functional vaccine antigens, antibodies, and other therapeutic proteins; as well as enzymes for industrial use, have been produced in microalgae (Scranton et al., 2015; Rosales-Mendoza, 2016a). The highest yields, observed thus far, were achieved in photosynthetic microalgae transformed at the chloroplast genome; with yields up to 3 mg/L culture (1.86% of Total Soluble Protein (TSP); Gimpel et al., 2015). Nonetheless, the development of transplastomic clones requires laborious construction of species-specific vectors and the system is unable to carry out complex post-translational modifications (e.g., glycosylation) or secrete the recombinant protein. In the case of nuclear expression, genetic engineering faces two challenges: the improvement of yields and the genetic stability of the transformed clones (Rosales-Mendoza, 2016b). In an effort to overcome these obstacles, UV mutagenesis has been used to generate *Chlamydomonas reinhardtii* strains with improved transgene expression (showing yields up to 0.2% TSP; Neupert et al., 2009; Kurniasih et al., 2016). Secretion approaches have also been implemented in these mutant strains, leading to yields up to 10 mg of secreted reporter protein per liter of culture (Lauersen et al., 2013).

Viral vectors have been widely applied in the expression of heterologous proteins in plants (Gleba et al., 2007; Lico et al., 2008; Chen et al., 2011; Dugdale et al., 2013; Salazar-González et al., 2015). For instance, the Magnifection system described 10 years ago became a powerful tool for the expression of recombinant proteins in higher plants via transient expression (Gleba et al., 2005, 2013; Marillonnet et al., 2005). In the case of microalgae, some viral elements have been used to innovate genetic engineering approaches. For instance, the picornaviral 2A sequence has allowed the expression of polycistrons (Rasala et al., 2012) while synthetic promoters have led to improved expression (Scranton et al., 2016). Surprisingly, the use of viral vectors has not been explored in algal biotechnology thus far. Herein the expression of biopharmaceuticals using a geminiviral vector was explored in the marine microalga *Schizochytrium* sp., which is an attractive species due to its ability to grow heterotrophically in marine water; thus not depending on photobioreactors. We used two antigen models for viral (glycoprotein 1, GP1, from *Zaire ebolavirus*) and bacterial (the B subunit of *Escherichia coli* heat-labile enterotoxin, LTB) diseases, which have high impact on the public health of developing countries. The viral vector-based expression system was functional and led to efficient expression of biopharmaceuticals.

## Materials and methods

### Vector design

An inducible vector was designed using the following elements: the *AlcR* gene and *AlcA* promoter from *Aspergillus nidulans*, the replication protein “Rep,” and the origin of replication “Ori” from the geminivirus *Ageratum enation virus*. The elements of the viral vector were placed in the following order: *Cauliflower mosaic virus 35S* promoter, *AlcR* gene, nopaline synthase terminator (nos), Ori, *AlcA* promoter, *Sma* I, *Bam*HI, and *Pst* I restriction sites, 35S terminator, Ori (repeated), *AlcA* promoter, and Rep protein ORF. A DNA fragment containing this array was synthetized by GenScript Inc. (New Jersey, USA) and sub-cloned in the binary vector pBI121 at the *Xba*I-*Sac* I sites through standard digestion-ligation procedures. A positive clone identified by restriction profile was used to perform sequencing and confirm sequence integrity; the construct was subsequently transferred into *Agrobacterium tumefaciens* (GV3101 strain) by electroporation (Cangelosi et al., 1991). A clone carrying the expression vector was confirmed by PCR and subsequently cultivated overnight in Luria Bertani broth at 25°C and 200 rpm to perform expression experiments.

### Microalga transient transformation

The *Schizochytrium* sp. strain 20888 was obtained from ATCC (USA), it was cultured at 25°C in modified seawater medium (5 g/L peptone, 1 g/L yeast extract, 0.2 g/L FeSO_4_, 15 g/L agar, and 35 g/L NaCl). For liquid cultures, the 679BY medium was used (1 g/L yeast extract, 1 g/L peptone, 5 g/L dextrose, and 35 g/L NaCl). Cultures were incubated at 150 rpm. Transformation was carried out by inoculating *Schizochytrium* sp. cultures (100 mL, OD_650nm_ = 0.7) with 1 mL of *A. tumefaciens* culture (OD_650nm_ = 1.0). The medium was supplemented with 100 μM acetosyringone. 16 h post-inoculation cefotaxime was added at a final concentration of 250 mg/L. The expression of the gene of interest in the transiently transformed microalgae was induced 20 h post-inoculation by the addition of 1 mL of absolute ethanol (1% of final concentration). Culture samples were collected before induction and 12, 24, and 48 h post-induction. Samples were stored at −80°C until further use.

### Inverse PCR

Circular DNA replicons were detected by inverse PCR using the following primer sets: for GP1, forward 5′CATCACCAAGATACCGGAGAAG and reverse 5′TTTAGTTTCCCAGAAGGCCC3′; for LTB, forward 5′CATTTCCCTCTTTCCAGCCAand reverse 5′TTATGGAGAAACTCGAGCTTGT 3′. Total DNA was isolated from *Schizochytrium* sp. cultures according to Dellaporta et al. (1983). PCR reaction mixtures (25 μL) contained 1 × PCR buffer, 100 ng DNA, 1.5 mM magnesium chloride, 2.5 U Taq DNA polymerase, 1 mM dNTPs, and 1 μM of the corresponding primer set. Cycling conditions were: 94°C for 5 min (initial denaturation); 35 cycles at 94°C for 30 s, 55°C for 30 s, 72°C for 120 s, and a final extension at 72°C for 10 min. PCR products were detected by electrophoresis in 1% agarose gels. The negative control consisted of 100 ng of DNA from WT *Schizochytrium* sp. culture.

### Protein analysis

The integrity of the *Schizochytrium*-made recombinant proteins was assessed by Western blot analysis. Culture samples were collected at different post-induction times (0, 12, 24, and 48 h) and protein extracts were obtained as follows: 20 mg of fresh biomass were resuspended in 200 μL of extraction buffer (750 mM Tris-HCl, pH 8, 15% sucrose, 100 mM β-mercaptoethanol, and 1 mM PMSF) and sonicated (4 pulses of 4 s with a 4 s delay in between) using an ultrasonic processor equipment at 24% amplitude (model GEX130PB). Samples were subsequently centrifuged at 8,000 rpm for 15 min and the supernatants were transferred to new microtubes. Total soluble protein concentration was determined in the extracts by the Lowry method and a volume corresponding to 80 μg of TSP (approximately 100 μL) was mixed with the same volume of 2 × reducing loading buffer, denatured by boiling for 5 min at 95°C, and subsequently subjected to SDS-PAGE analysis. Gels were blotted onto 0.45 μm Bio-Rad nitrocellulose membranes (http://www.bio-rad.com). Protein transfer was performed using a TV100-EBK Electroblotter (AlphaMetrix Biotech, GER) for 1 h at 150 V in a methanol-based transfer buffer. After blocking with 5% fat-free milk (Carnation, Nestle) dissolved in PBS-0.1% Tween, the blots were incubated with mouse anti-sera (1:200 dilution) against either LTB or GP1; which were produced in mice as previously described (Orellana-Escobedo et al., 2015). The blots were washed and incubated with a goat horseradish peroxidase-conjugated secondary anti-IgG mouse antibody (1:2,000 dilution, Sigma) for 2 h at room temperature. Antigen detection was revealed by incubating blots with the SuperSignal West Dura solution following the instructions from the manufacturer (Thermo Scientific, http://www.thermoscientific.com) and exposing the film to standard procedures. LTB and GP1 proteins (250 ng) purified from recombinant *E. coli* were used as positive controls.

For quantitative ELISA, a protocol described elsewhere was applied (Ríos-Huerta et al., 2017). Briefly, protein extracts were obtained as described above and diluted with 0.2 M carbonate buffer (pH = 9.6). The diluted sample was added to an ELISA plate for protein adsorption at 4°C. After blocking with 5% fat-free dry milk solution for 2 h at room temperature, the plates were incubated with either anti-LTB (1:500 dilution) or anti-GP1 mouse serum (1:1,000 dilution) overnight at 4°C. The plates were subsequently incubated with a rabbit horseradish peroxidase-conjugated anti-mouse IgG (1:2,000 dilution) for 2 h at 25°C. A colorimetric reaction was induced by addition of the ABTS substrate solution (0.6 mM 2,2′-azino-bis(3-ethylbenzothiazoline-6-sulphonic acid), 0.1 M citric acid, pH = 4.35, and 1 mM H_2_O_2_), after 30 min of incubation at 25°C OD values were measured at 405 nm. Standard curves were constructed using pure LTB or GP1 to estimate expression levels.

## Results

### The pAlgevir vector is delivered and replicated in microalgae

**Figure 1:**
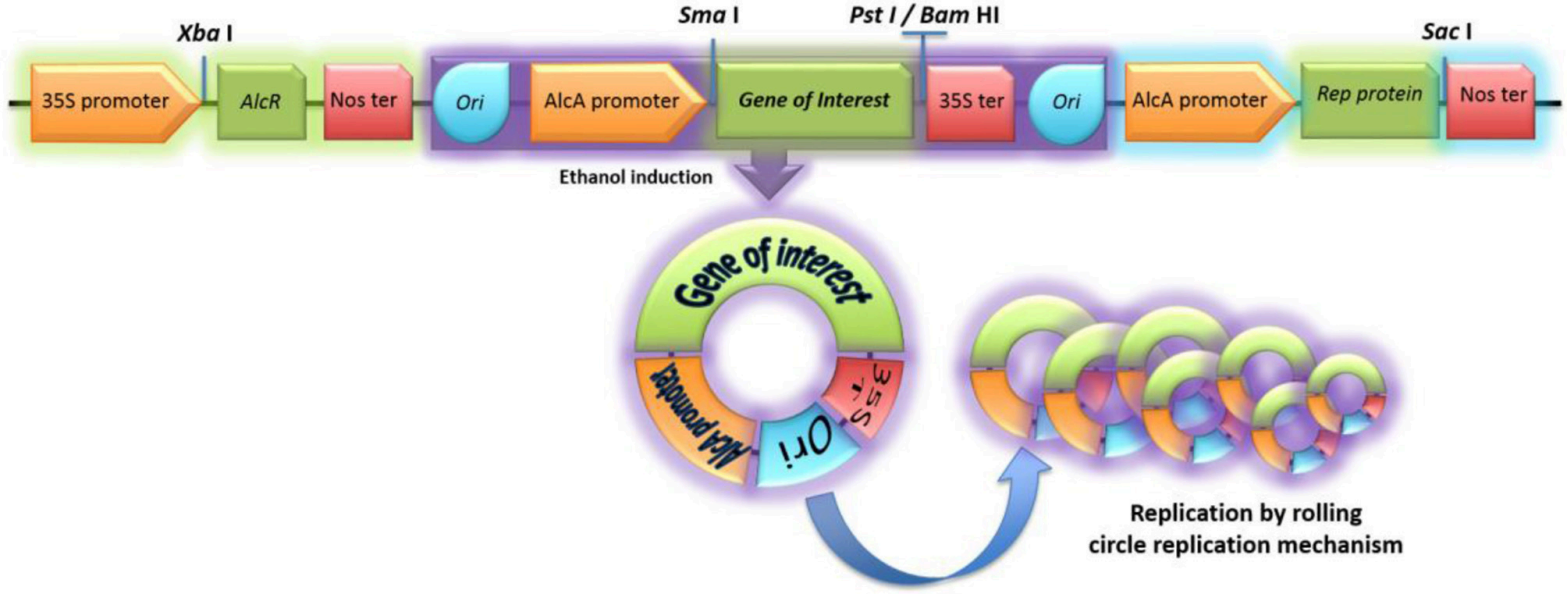
Physical map of the pAlgevir vector. The vector mediates the ethanol-inducible expression of the target protein through the AlcR transcription factor, which is constitutively expressed under the control of the *Cauliflower mosaic virus 35S* promoter and upon ethanol presence activates the *AlcA* promoter. The *AlcA* promoter and AlcR belong to *Aspergillus nidulans*. The Rep protein leads to replication of the expression cassette flanked by Ori elements.

Gene synthesis and molecular cloning techniques allowed the construction of the pAlgevir vector; whose physical map is shown in Figure 1 while the restriction profile and sequence are shown in Figures S1, S2, respectively. The *AlcR* gene from *A. nidulans* is under the control of the *Cauliflower mosaic virus 35S* promoter and the nos terminator, whereas the Rep protein is under the control of the *AlcA* inducible promoter from *A. nidulans* and the nos terminator. The gene of interest, which is inserted into the *Sma*I at the 5′ end and either *Bam*HI or *Pst*I sites at the 3′ end, is under the control of the inducible *AlcA* promoter and the 35S terminator. Upon ethanol induction, the transcription factor AlcR is activated leading to the expression of the gene of interest and the Rep protein. The latter acts on the Ori elements and mediates the generation and replication of circular DNA molecules carrying the expression cassette for the biopharmaceutical. The obtained restriction profile for the pAlgevir vector is shown in Figure S1.

### Algevir allows efficient expression of viral and bacterial antigens

**Figure 2:**
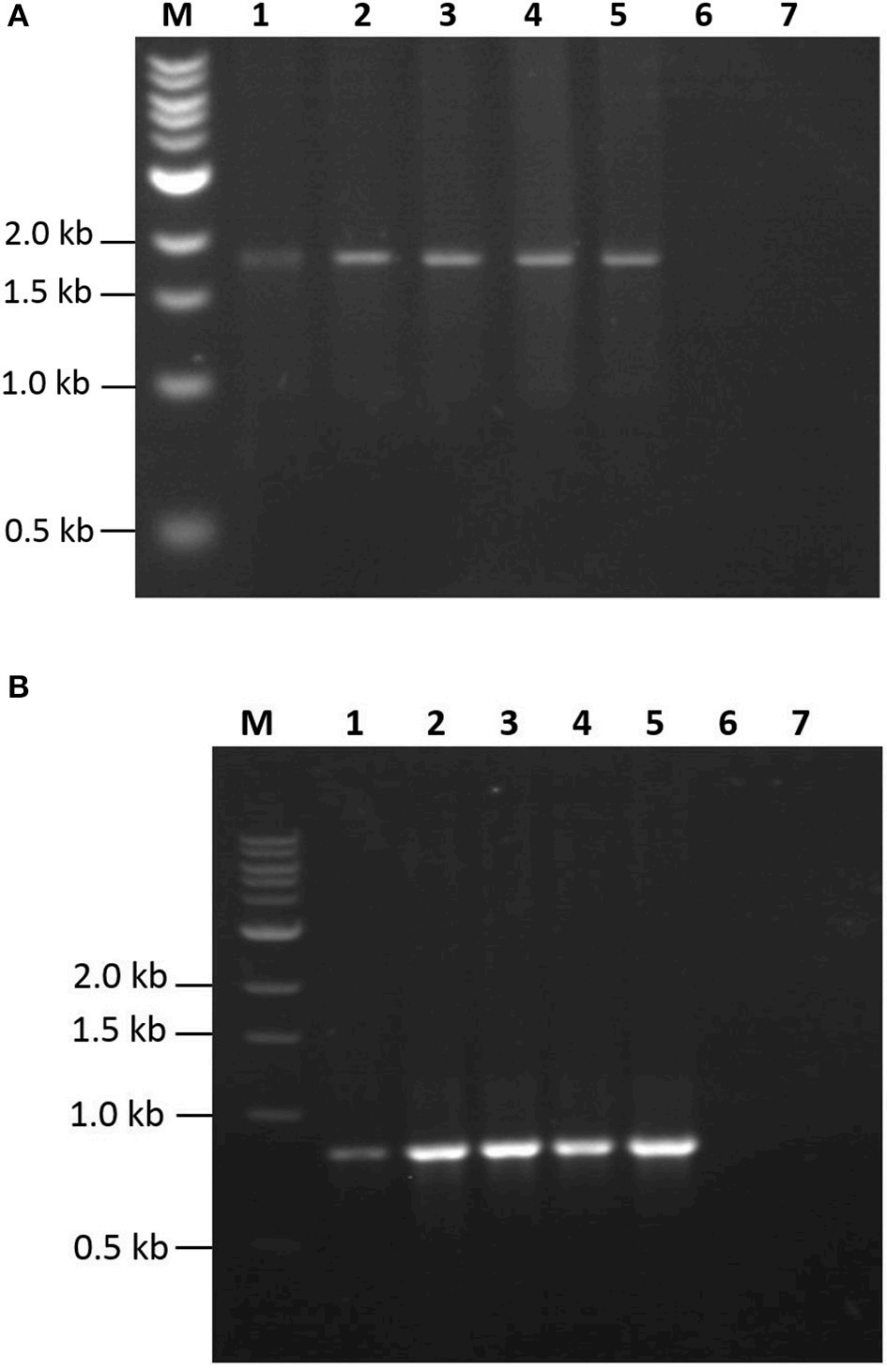
Detection of replicons by inverse PCR. Total DNA samples from *Schizochrytrium* sp. transiently transformed with pAlgevir-GP1 **(A)** and pAlgevir-LTB **(B)** vectors were analyzed by PCR using primers to detect circular replicons generated by Rep. Lanes: M, 1 Kb ladder (New England, biolabs); 1–5, DNA samples from *Schizochrytrium* sp. cultures transiently transformed with *A. tumefaciens* carrying the corresponding pAlgevir vector at 0, 12, 24, 48, and 72 h post-induction, respectively; 6: DNA sample from wild type *Schizochrytrium* sp. culture; 7, negative control (water). Expected amplicons for the detection of GP1 and LTB replicons are 1,800 and 918 bp in length, respectively.

**Figure 3:**
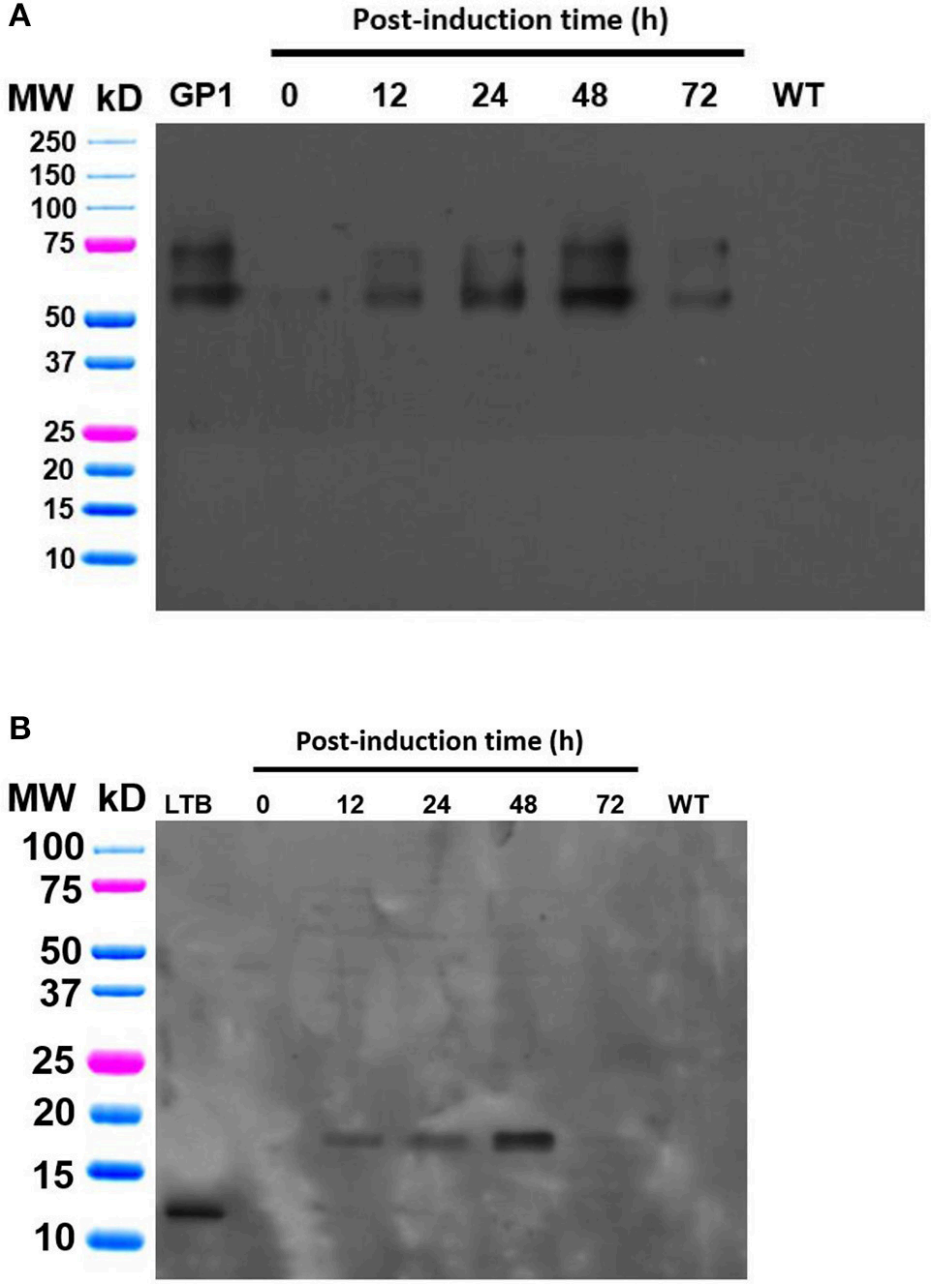
Immunodetection of the GP1 and LTB recombinant proteins produced in *Shizochrytrium* sp. through the Algevir system. *Schizochrytrium* sp. cultures were transiently transformed with *A. tumefaciens* carrying the pAlgevir vector. Total soluble proteins were extracted and subjected to Western blot analysis labeling with anti-sera against GP1 **(A)** or LTB **(B)**. Positive controls consisted of 250 ng of either GP1 or LTB.

**Figure 4:**
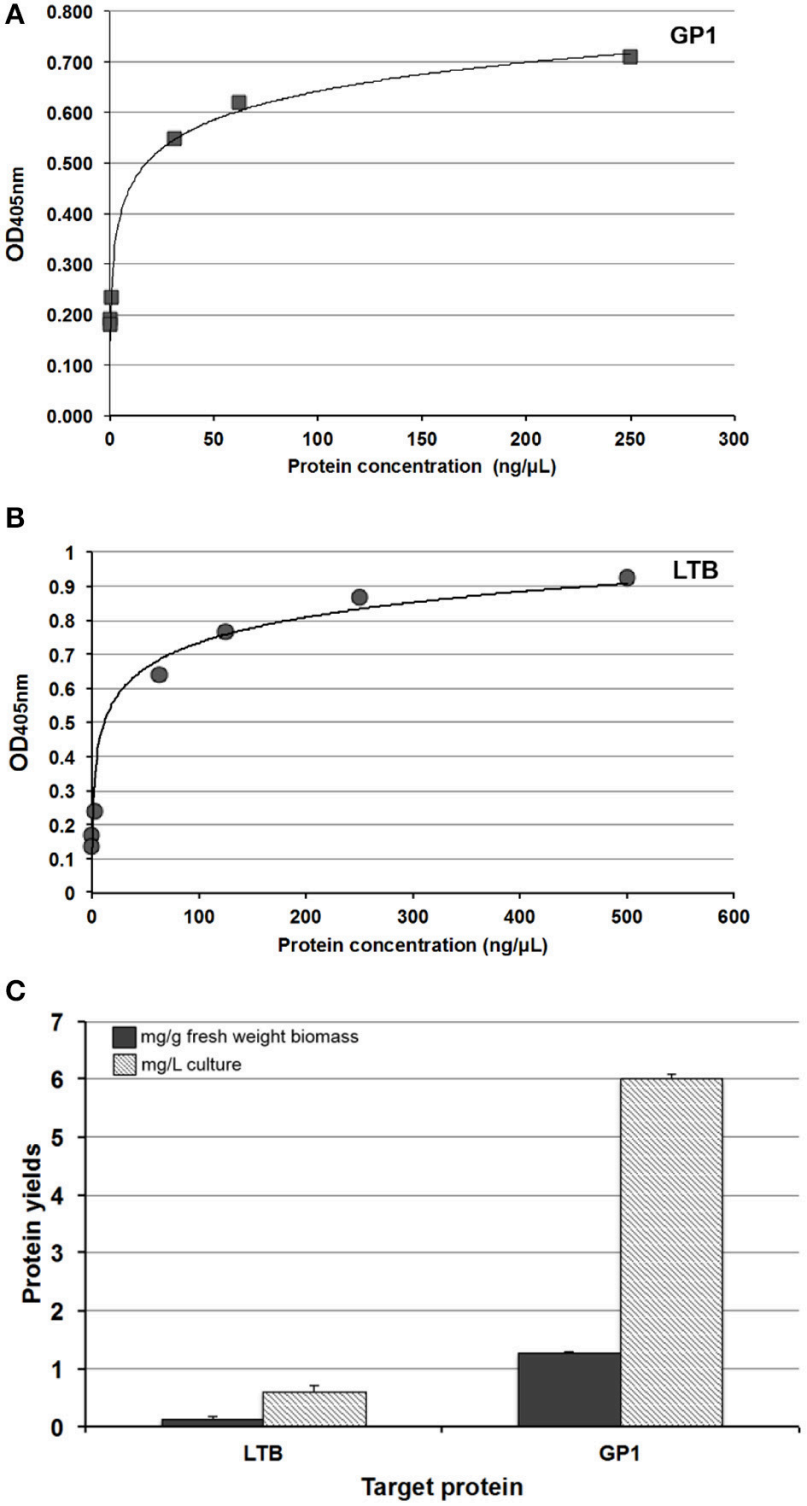
Yields of the recombinant proteins produced in *Shizochrytrium* sp. with the Algevir system. Quantitative ELISA was performed to determine the accumulation levels of the *Shizochrytrium* sp.-made proteins 48 h post-induction. Pure proteins were used to construct standard curves for GP1 **(A)** and LTB **(B)**. Accumulation levels are expressed as milligrams of recombinant protein per gram of fresh microalgae biomass or milligrams of recombinant protein per liter of microalgae culture **(C)**. The presented values are the means of triplicates ± standard deviation.

Expression studies were conducted to evaluate the efficiency of the Algevir system. *Schizochytrium* sp. cultures were inoculated with recombinant agrobacteria and induced with ethanol 20 h post-inoculation, with the subsequent analysis to determine vector replication and recombinant protein yields at different post-induction times. We first investigated the presence of DNA replicons through inverse PCR analysis to prove the expression of functional Rep leading to replication of the expression cassette. In this approach PCR primers landing in the expression cassette flanks but at a divergent position allow the amplification of circular molecules only, which are generated by rolling circle replication; whereas lineal forms of the expression cassette are not amplified. Transformed and induced *Schizochytrium* sp. cultures showed a positive result at any studied time point, indicating that the Algevir plasmid mediates the generation of replicons by rolling circle replication (Figure 2). The amplification was also detected at the pre-induction phase, which suggested a basal transcription of the Rep protein before ethanol induction. In terms of recombinant protein production, *Schizochytrium* sp. showed a minimum level of recombinant protein at the pre-induction phase. The recombinant protein levels dramatically increased upon induction, reaching the highest levels 48 h post-induction (Figure 3). According to an ELISA, the recombinant protein yields 48 h post-induction reached values up to 1.25 mg/g fresh weight (FW) for GP1 (6 mg/L of culture) and 0.12 mg/g FW (0.6 mg/L of culture) for LTB (Figure 4). Quantitative ELISA analysis confirmed that 48 h post-induction was the time point with maximum yields whereas shorter or extended post-induction times showed significantly lower protein yields (data not shown).

## Discussion

The use of viral vectors to produce recombinant proteins in algae has not been explored thus far. Herein, the Algevir system was developed as an attractive approach for the transient expression of recombinant proteins in microalgae using the replication elements from the begomovirus *Ageratum enation virus*. For the design of the Algevir vector we adopted an inducible vector comprising the *AlcA* promoter, which has a fungal origin and it is induced by ethanol in a mechanism mediated by the AlcR protein.

To assess the potential of the Algevir system we selected two relevant biopharmaceuticals: GP1 from *Z. ebolavirus*, which is a complex viral glycoprotein implicated in the development of subunit vaccines against Ebola since it induces neutralizing humoral responses (Sridhar, 2015); and a bacterial antigen with relevance in vaccination against enteric diseases (Zhang and Sack, 2015). These antigenic proteins were successfully expressed in *Schizochytrium* sp., revealing a robust capacity of the Algevir system for the expression of both bacterial and complex viral glycoproteins that retained their antigenicity. The maximum expression levels were attained for GP1 2 days post-induction, reaching a maximum value of 6 mg/L. Under our conditions the protein yields decreased at 72 h post-induction, since replicons are still detected at this time point; this decrease is most likely due to a lower transcriptional activity associated to a decrement in ethanol levels.

In the field of biopharmaceuticals production using recombinant algae, the most productive systems for expressing heterologous proteins comprise the following: (i) Yields up to 3 mg/L were achieved by Gimpel et al. (2015) for the Bovine Milk Amyloid A protein following a chloroplast expression approach. Nonetheless, chloroplast expression restricts the system to proteins not requiring glycosylation (no evidence has been reported on these post-translational modifications occurring in the chloroplast of microalgae). (ii) The recently reported nuclear expression approaches led to yields up to 15 mg/L under the modality of recombinant protein secretion to the culture medium, using either synthetic glycomodules (tandem serine and proline repeats) that improve secretion or a mutant strain that efficiently expresses heterologous genes (Lauersen et al., 2013; Ramos-Martinez et al., 2017). However, these yields correspond to evaluations using reporter genes and not complex biopharmaceuticals. (iii) Recombinant hemagglutinin proteins from the influenza virus were expressed in the microalga *Schizochytrium* sp. in a secretion modality reaching yields up to 20 mg/L. It should be considered that this system is conceived for the production of parenteral vaccines through purification of the antigen from culture supernatants (Bayne et al., 2013).

When compared to the previous systems, the Algevir system is considered attractive since it possesses features that overcome some of the limitations showed by the existing expression approaches. First, nuclear expression allows accessing complex cellular machinery to perform glycosylation and other post-translational modifications. In contrast to secretion approaches, the intracellular accumulation of the antigens allows using the whole cell as vaccine delivery vehicle; which is relevant since it can serve as a biocapsule. For instance, *Schizochytrium* sp. produces several endogenous compounds that could exert immunomodulatory effects such as polyunsaturated fatty acids (Bragaglio et al., 2015). Second, Algevir is based on the inducible expression of the target protein, which offers short production times (1 week at the most) and the particular advantage of allowing the expression of proteins exerting toxic effects in the host since biomass production is dissociated from the recombinant protein expression phase. Third, since Algevir is based on transient expression; the problems related to the generation of stable transformants that are prone to genetic instability are avoided (Rosales-Mendoza, 2016b). Therefore, Algevir is a versatile system offering the advantages of transient expression (short production time and high yields) that are ideal for the production of vaccines in response to epidemics.

The Algevir system was initially tested with the marine microalgae *Schizochytrium* sp., which is grown heterotrophycally and currently produced at an industrial scale; thus the adoption and scale-up of this technology will be greatly facilitated (Qu et al., 2013; Song et al., 2015). In conclusion, the Algevir system is a robust approach for producing recombinant proteins in microalgae that will open a new path in algal biotechnology.

## Author contributions

BB performed most of the experiments. BB and SR carried out the experimental design, data analysis and wrote the manuscript. EM conducted protein detection analysis. CA and OG contributed to the data analysis. All authors discussed the results, read and approved the final version of the manuscript.

### Conflict of interest statement

The authors declare that the research was conducted in the absence of any commercial or financial relationships that could be construed as a potential conflict of interest. The work presented in this report is the subject of a pending patent.
