# Supplementary material for: Algevir: An Expression System for Microalgae Based on Viral Vectors
Source: Front Microbiol. 2017 Jun 30;8:1100. doi: 10.3389/fmicb.2017.01100 (PMC5491637; doi:10.3389/fmicb.2017.01100)
Supplement: Supplementary file 1 [file DataSheet1.docx]

**Supplementary material**

**Figures legends**

**Figure S1.** Restriction profile analysis of the pAlgevir vector. Lanes: M, 1 kb DNA ladder (New England Biolabs); 1, undigested pAlgevir vector; 2, digested pAlgevir vector using *Xba* I and *Sac* I. *Xba* I*/Sac* I digestion releases a 5 kb fragment corresponding to *AlcR* to *Rep* gene.

**Figure S2.** Sequence of the expression cassettes from the pAlgevir vector.

**Figures**

**Fig. S1**

Fig. S2

TCTAGAATGGGTCAACAGCTTGAAAAGCCTCTCTCTCCATTATCATTCCAGGCTGTTCTC

TTACCTCCACATTCTCCTAATACTGATGATCTCATTAGAGAGTTAGAAGAGCAAACTACA

GATCCAGATTCAGTGACAGATACCAACAGTGTTCAACAGGTGGCTCAGGATGGAAGTCTT

TGGTCTGATAGACAAAGTCCTCTTTTGCCAGAAAATTCTTTGTGTATGGCTTCAGATAGT

ACAGCAAGAAGATATGCTAGGTCTACTATGACAAAGAACCTTATGAGAATCTACCATGAT

TCTATGGAAAATGCTCTTTCATGTTGGTTGACTGAGCACAACTGCCCTTATAGTGATCAA

ATTTCTTACCTTCCTCCAAAACAAAGGGCTGAATGGGGACCAAATTGGTCTAACAGAATG

TGTATAAGAGTTTGCAGGTTGGATAGAGTGTCTACATCACTCAGAGGTAGGGCATTATCT

GCTGAAGAGGATAAGGCTGCTGCTAGGGCACTCCATCTTGCTATTGTTGCATTTGCTTCA

CAGTGGACTCAGCACGCACAAAGAGGAGCTGGTCTTAATGTGCCTGCTGATATAGCAGCT

GATGAAAGGTCTATTAGAAGGAATGCTTGGAACGAGGCAAGACATGCTTTGCAGCACACC

ACTGGTATTCCTTCTTTTAGAGTTATTTTCGCTAACATTATCTTCTCACTTACACAGAGT

GTGTTGGATGATGATGAACAACATGGAATGGGTGCTAGGCTTGATAAACTCTTAGAAAAT

GATGGTGCACCTGTTTTTCTCGAGACAGCTAACAGACAGTTATATACCTTTAGGCACAAG

TTCGCAAGAATGCAAAGAAGGGGAAAGGCTTTTAATAGACTTCCAGGAGGTTCAGTTGCA

AGTACTTTTGCTGGTATATTCGAAACCCCTACTCCAAGTTCTGAGTCACCTCAACTTGAT

CCTGTTGTGGCTAGTGAAGAGCATAGATCCACTCTTAGTCTTATGTTTTGGCTTGGTATC

ATGTTCGATACTTTGTCTGCTGCTATGTACCAGAGACCTCTTGTTGTGTCTGATGAGGAT

TCACAAATATCAAGTGCATCACCTCCAAGAAGGGGAGCTGAAACACCTATTAATTTGGAT

TGTTGGGAGCCTCCTAGACAGGTTCCATCAAACCAAGAAAAAAGTGATGTGTGGGGAGAT

TTGTTTCTTAGAACAAGTGATTCTTTGCCTGATCATGAATCTCACACCCAGATTTCACAA

CCTGCTGCTAGATGGCCATGCACTTATGAGCAAGCTGCTGCTGCTTTGTCTTCAGCTACA

CCTGTTAAGGTGCTTTTGTATAGAAGGGTTACTCAACTCCAGACACTCTTATACAGGGGA

GCATCTCCAGCTAGACTCGAAGCAGCTATCCAGAGAACTTTATATGTGTACAATCATTGG

ACAGCAAAATATCAGCCTTTCATGCAAGATTGTGTTGCTAACCACGAGCTTTTGCCATCA

AGAATCCAATCTTGGTATGTTATCCTCGATGGTCATTGGCACTTAGCAGCTATGCTCTTA

GCTGATGTTCTCGAATCTATAGATAGGGATTCATACAGTGATATTAATCATATTGATCTT

GTTACTAAACTTAGATTGGATAACGCACTCGCTGTGTCAGCATTAGCTAGGAGTTCTCTT

AGAGGACAAGAACTTGATCCTGGAAAGGCTTCTCCAATGTATAGACATTTTCACGATTCA

CTTACCGAAGTTGCTTTCTTGGTGGAGCCGTGGACTGTTGTGCTTATCCATTCTTTTGCT

AAGGCAGCTTACATACTTTTGGATTGTCTTGATCTTGATGGACAGGGTAACGCATTGGCT

GGATACCTTCAATTGAGACAAAACTGTAACTACTGCATCAGGGCTCTTCAATTCTTGGGT

AGAAAATCTGATATGGCAGCATTGGTGGCTAAGGATTTGGAAAGGGGACTCAACGGTAAA

GTGGATTCATTCTTGTAAGATCGTTCAAACATTTGGCAATAAAGTTTCTTAAGATTGAAT

CCTGTTGCCGGTCTTGCGATGATTATCATATAATTTCTGTTGAATTACGTTAAGCATGTA

ATAATTAACATGTAATGCATGACGTTATTTATGAGATGGGTTTTTATGATTAGAGTCCCG

CAATTATACATTTAATACGCGATAGAAAACAAAATATAGCGCGCAAACTAGGATAAATTA

TCGCGCGCGGTGTCATCTATGTTACTAGATCGGGCACGTGGGTACCGTTGACTTAGTCAA

TCGGTACTCAGCTCTAGTCTTATGTCAATTGGTGATCAGTACTCAATATATAGTGAGTAC

CTAATGGCATTATTCGTAATTTTGAAAAGAAATTCAAAATTCAAATTTGAAATCCAAAAG

CGGCCATCCGTATAATATTACCGGATGGCCGCGATTTTTTAAAGTGGTCCCCCCAGTGCA

CTAACTGACAAAGACATGTCCACCAATCTAAATCGTCGCTCAAAGCTAAATTGTTTTGTG

GTCCCTTATTTAAACTTGCTCATCAAGTAGTGCACTCCGCACTCCTGCACGCGCAGCTCG

GGATAGTTCCGACCTAGGATTGGATGCATGCGGAACCGCACGAGGGCGGGGCGGAAATTG

ACACACCACTCCTCTCCACGCACCGTTCAAGAGGTACGCGTATAGAGCCGTATAGAGCAG

AGACGGAGCACTTTCTGGTACTGTCCGCACGGGATGTCCGCACGGAGAGCCACAAACGAG

CGGGGCCCCGTACGTGCTCTCCTACCCCAGGATCGCATCCCCGCATAGCTGAACATCTAT

ATAAGGAAGTTCATTTCATTTGGAGAGGAACTAGTCCCGGGATGCTGCAGGGATCCAGAT

CTTAATTCGGGGGATCTGGATTTTAGTACTGGATTTTGGTTTTAGGAATTAGAAATTTTA

TTGATAGAAGTATTTTACAAATACAAATACATACTAAGGGTTTCTTATATGCTCAACACA

TGAGCGAAACCCTATAGGAACCCTAATTCCCTTATCTGGGAACTACTCACACATTATTAT

GGAGAAACTCGAGCTTGTCGATCGACAGATCCGAGGTTGACTTAGTCAATCGGTACTCAG

CTCTAGTCTTATGTCAATTGGTGATCAGTACTCAATATATAGTGAGTACCTAATGGCATT

ATTCGTAATTTTGAAAAGAAATTCAAAATTCAAATTTGAAATCCAAAAGCGGCCATCCGT

ATAATATTACCGGATGGCCGCGATTTTTTAAAGTGGTCCCCCCAGTGCACTAACTGACAA

AGACATGTCCACCAATCTAAATCGTCGCTCAAAGCTAAATTGTTTTGTGGTCCCTTATTT

AAACTTGCTCATCAAGTAGTGCACTCCGCACTCACGTGAAGCTTCGCAGCTCGGGATAGT

TCCGACCTAGGATTGGATGCATGCGGAACCGCACGAGGGCGGGGCGGAAATTGACACACC

ACTCCTCTCCACGCACCGTTCAAGAGGTACGCGTATAGAGCCGTATAGAGCAGAGACGGA

GCACTTTCTGGTACTGTCCGCACGGGATGTCCGCACGGAGAGCCACAAACGAGCGGGGCC

CCGTACGTGCTCTCCTACCCCAGGATCGCATCCCCGCATAGCTGAACATCTATATAAGGA

AGTTCATTTCATTTGGAGAGGAACTAGTATGGCAGCACCTAATAGATTCAAGATTAACGC

TAAAAATTACTTTCTGACTTACCCTAAATGTTCACTCACAAAAGAAGAGGCACTGTCCCA

GCTTTTGAACCTCCAAACTCCTACAAATAAGAAGTATATTAAGATTTGCAGAGAGCTGCA

TGAAGATGGTTCTCCACATCTTCATGTTTTGATTCAGTTTGAGGGCAAGTACCAGTGCAA

GAATCAAAGATTTTTCGATCTTGTCAGTCCTAACAGGTCCGCTCATTTCCACCCAAATAT

TCAGAGGGCAAAGTCTTCAACAGATGTCAAGTCTTATATCGATAAAGATGGAGATACCTT

GGAGTGGGGTGAATTTCAAGTTGATGGCAGATCAGCTAGGGGAGGACAGCAAACCGCAAA

CGATGCTGCACCTGAGGCTCTTAATGCAGGAAGTAAAGAAGCAGCTATGGCTATTATCAG

AGAGAAGTTGCCTGAAAAGTTTATTTTCCAATACCATAATCTCAACTCCAATCTGGATAG

GATCTTCACACCTCCACTCGAGGTTTACGTGAGCCCTTTTCTGAGTTCCAGCTTCGATCA

GGTTCCAGAGGAACTCGAGGAATGGGTGTCTGAGAATGTCATGGTTGCAGCTGCAAGACC

TCTTAGGCCACAATCAATTGTTATCGAAGGAGATAGTAGAACCGGAAAGACTATGTGGGC

TAGGTCACTTGGGCCTCATAACTACTTGTGCGGACACCTCGATCTGAGCCCAAAAGTGTA

TTCTAATAACGCTTGGTACAATGTGATTGATGATGTCGATCCTCATTATCTTAAGCACTT

CAAGGAATTCATGGGAGCACAGAGAGATTGGCAAAGTAATACTAAGTACGGTAAACCTGT

TCAGATCAAGGGCGGGATTCCAACAATCTTTCTTTGTAACCCTGGACCAAATTCTTCATA

TAAGGAGTTCTTGGATGAGGAGAAGAATAACGCTCTCAGGAACTGGGCTCTGAAAAATGC

AATTTTTGTGACTCTTGAAGGCCCATTGTACTCTGGGTCAAACCAATCAGCAGCACAGGC

AAGCCAAGAGGGAGATGAAGCAAGCACATGTTAATAAGAGCTC
